# Upper Vault Septal Anatomy and Short Nasal Bone Syndrome: Implications for Rhinoplasty

**Published:** 2018-10-04

**Authors:** Arian S. Mowlavi, Tiffany L. Chamberlain, Astrid Melgar, Armin Talle, Sean Saadat, Soheil Sharifi-Amina, Bradon J. Wilhelmi

**Affiliations:** ^a^Cosmetic Plastic Surgery Institute, Laguna Beach, Calif; ^b^Western University of Health Sciences, Pomona, Calif; ^c^Department of Biological Sciences, University of California, Irvine; ^d^The George Washington University School of Medicine and Health Sciences, Washington, DC; ^e^Riverside Community Hospital, Riverside, Calif; ^f^Hiram C. Polk Jr MD Department of Surgery, University of Louisville School of Medicine, Louisville, Ky

**Keywords:** short nasal bone syndrome, rhinoplasty, saddle nose deformity, nasal mid-vault collapse, nasal depression

## Abstract

**Introduction:** This is a diagnostic study that investigates the clinical significance between patients with short and long nasal bones and the variation in upper septal composition that would delineate propensity for middle vault collapse. **Methods:** Computed tomographic scans of 16 female patients undergoing evaluation with sinus films were analyzed. Two measurements were taken from each scout image: nasal bone length and total nasal length. Patient scans were separated into 2 groups; patients whose nasal bone length was less than one-half their total nasal length were defined as patients with “short nasal bone” (n = 8), and those with nasal bones longer than one-half the length of their noses were defined as patients with “long nasal bone” (n = 8). **Results:** Key differences were identified between patients with short and long nasal bones. Total septal area in the upper vault was decreased in the short nasal bone group relative to that of the long nasal bone group (5.7 ± 0.6 cm^2^ vs 8.1 ± 1.0 cm^2^, *P* = .002). This was mainly the result of the decreased ethmoid bone component in the short nasal bone group when compared with the long nasal bone group (1.6 ± 0.6 cm^2^ vs 3.2 ± 0.8 cm^2^, *P* = .007).

Saddle nose deformity is a condition in which a loss of cartilaginous support causes the nasal mid-vault to collapse.^1^ This condition causes a highly visible “saddle,” or depression, to form on the nasal dorsum. The condition results in compromise not only of the functional airway, as the loss of mid-vault support compromises the nasal airway, but also of the aesthetic contour, as the nasal dorsum becomes visibly concave.^2^ While a handful of pathologic conditions can cause saddle nose deformity, most reported incidences are the result of trauma or prior surgery to the nose.^1^

A predisposition for saddle nose deformity following rhinoplasty has been observed in patients with nasal bones that span less than one-third to one-half of the total length of the nose; these patients are said to have short nasal bones.^3^ The propensity for saddle nose deformity in patients with short nasal bone was presumed to result from a longer and consequently more vulnerable cartilaginous septal component, with less support from their relatively short nasal bones.^3^^,^^4^ Patients with long nasal bones have a larger ratio of bone to cartilage in their nasal mid-vault, which is felt to lend more rigid support and resistance to buckling following trauma or surgery.

Findings by Mau et al^5^ suggest that the strength of the nasal mid-vault, and consequently its resistance to saddle nose deformity, is reliant upon the stability of the nasal septum, in particular the variability in composition of the bone to cartilaginous makeup.^5^ We hypothesized whether the findings of Mau et al may shed light on the propensity for mid-vault compromise of patients with short nasal bone; could difference in septal cartilage and bony junction composition rather than traditionally believed difference in longitudinal span of cartilage be the real culprit? In this radiologic study, we take a closer look at the bony component of the upper septal support wall in patients with short versus long nasal bones to elucidate the relationship between short nasal bones and their increased susceptibility for nasal mid-vault collapse.

## METHODS

**Figure 1:**
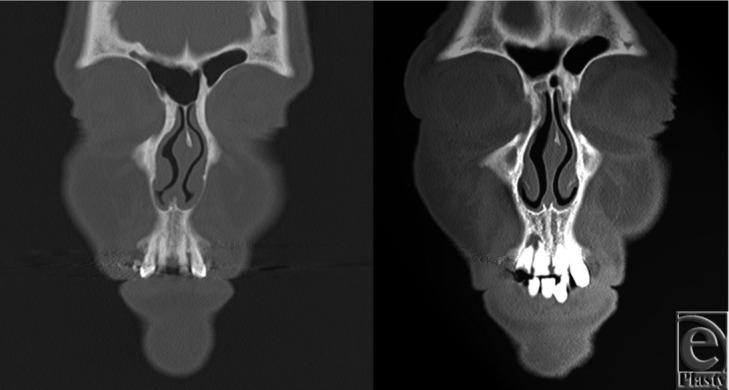
These scout computed tomographic scans were evaluated to determine the nasal bone length and total nasal length of each patient. Patients were then divided into a short nasal bone group (nasal bone length to total nasal length ratio < 1/2) (left) and a long nasal bone group (nasal bone length to total nasal length ratio > 1/2) (right).

**Figure 2:**
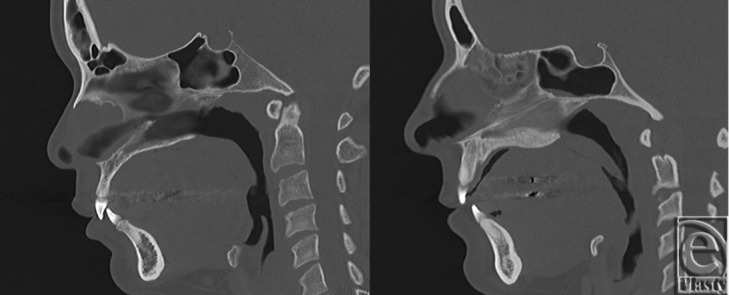
These coronal computed tomographic scans demonstrate the variable bony and cartilaginous composition of the nasal septum in the upper vault. Maximum ethmoid bone height was decreased in the short nasal bone group (left) when compared with the long nasal bone group (right).

Computed tomographic scans of 16 female patients undergoing evaluation with sinus films were analyzed. Two measurements were taken from each scout image: nasal bone length and total nasal length (Fig 1). Patient scans were separated into 2 groups; those patients whose nasal bone length was less than one-half their total nasal length were defined as patients with “short nasal bone” (n = 8), and those with nasal bones longer than one-half the length of their noses were defined as patients with “long nasal bone” (n = 8). We then evaluated the bone versus cartilage composition of the septal support structure in the upper vault underlying the nasal bones of each patient: the perpendicular plate of the ethmoid bone, the vomer bone, and the quadrangular cartilage (Fig 2). For each patient, we took the following steps: (1) We created a series of 3-mm coronal CT scans of increasing depth along the anteroposterior axis. (2) We calculated the area of each component (ethmoid bone, vomer bone, and quadrangular cartilage). (3) We recorded the maximum height observed of each of these structures. (4) The combined area of the vomer and ethmoid bone components was expressed as the total bony component area, and this value summed with the area of the quadrangular cartilage was expressed as the total septal area.

These values were averaged within the short and long nasal bone groups. All values were expressed as a mean ± standard deviation, and the data were analyzed for statistical significance using Student's *t* test.

## RESULTS

We identified key differences between patients with short and long nasal bones. Total septal area in the upper vault was decreased in the short nasal bone group relative to that of the long nasal bone group (5.7 ± 0.6 cm^2^ vs 8.1 ± 1.0 cm^2^, *P* = .002) (Table 1). This was mainly the result of the decreased ethmoid bone component in the short nasal bone group when compared with the long nasal bone group (1.6 ± 0.6 cm^2^ vs 3.2 ± 0.8 cm^2^, *P* = .007). Similarly, maximum ethmoid bone height was notably lower in the short nasal bone group than in the long nasal bone group (1.9 ± 0.6 cm vs 2.7 ± 0.1 cm, *P* = .010) (Fig 2).

## DISCUSSION

Postoperative complications following rhinoplasty are relatively common—one study analyzing 153 secondary rhinoplasties identified 91 cases of mid-vault collapse.^6^ Previous studies proposed several explanations for this incidence, though none were conclusive; the prevailing belief was that the longer and consequently more vulnerable septal cartilage component over the mid-vault put these patients at a higher risk of saddle nose deformity.^1^^,^^5^ Using an ex vivo model, Mau et al^5^ demonstrated that both frontal and lateral forces applied to the dorsal septum most often resulted in breakage at the bony cartilaginous junction. In fact, preservation of an increased bony cartilaginous junction by preserving a triangular cephalad-cartilaginous septal-ethmoid component decreased the likelihood of breakage.^5^ On the basis of this study, we decided to study the upper vault contribution to the septal composition.^7^

We did not find any significant difference between the size of upper vault septal cartilage of patients with short and long nasal bones. Instead, we demonstrated that patients with short nasal bone possess a decreased ethmoid component of the upper vault nasal septum. This discrepancy in the upper vault anatomy supports our observation that patients with long nasal bone may in fact be protected against septal failure by virtue of their generous ethmoid bone component, which allows for an increased area of contact at the bony cartilaginous junction.

Our study suggests that patients with short nasal bone possess an upper vault nasal septum that differs in one key characteristic: it is composed of a reduced ethmoid bone component but similar quadrangular cartilage and vomer bone components. Although further studies must be performed, we hope that our findings of upper nasal septal anatomy will provide insight into the propensity of saddle nose deformity in patients with short nasal bone that relates to a smaller ethmoid cartilaginous junction rather than merely a longer mid-vault cartilaginous span.

## Figures and Tables

**Table 1:** Analysis of bone versus cartilaginous composition of the upper vault nasal septum in patients with short versus long nasal bones

	Short nasal bone	Long nasal bone	*P*^a^
Age, y	41 ± 7	34 ± 9	.188
Nasal length, cm	3.9 ± 0.4	4.1 ± 0.3	.413
Nasal bone length, cm	1.6 ± 0.2	2.1 ± 0.2	**.008**
Nasal bone/nasal length	0.39 ± 0.04	0.54 ± 0.03	**<.001**
Total septal area, cm^2^	5.7 ± 0.6	8.1 ± 1.0	**.002**
Ethmoid bone area, cm^2^	1.6 ± 0.6	3.2 ± 0.8	**.007**
Quadrangular cartilage area, cm^2^	3.8 ± 0.6	4.4 ± 0.6	.117
Vomer bone area, cm^2^	0.3 ± 0.3	0.5 ± 0.3	.340
Total bone component area, cm^2^	1.9 ± 0.6	3.7 ± 1.1	**.013**
Maximum total septal height, cm^2^	4.5 ± 0.3	4.9 ± 0.3	.124
Maximum ethmoid bone height, cm	1.9 ± 0.6	2.7 ± 0.1	**.010**
Maximum septal cartilage height, cm	2.7 ± 0.5	2.5 ± 0.3	.424
Maximum vomer bone height, cm	0.4 ± 0.3	0.6 ± 0.3	.345

^a^ Bold values indicate *P* > .05.
